# Supplementary material for: Targeted inCITE-Seq Analysis Identifies the Loss of Nuclear TDP-43 in Endothelium as a Mediator of Blood Brain Barrier Signaling Pathway Dysfunction in Neurodegeneration
Source: bioRxiv. 2023 Dec 14:2023.12.13.571178. Preprint. [Version 1] doi: 10.1101/2023.12.13.571178 (PMC10760156; doi:10.1101/2023.12.13.571178)
Supplement: Supplement 3 [file NIHPP2023.12.13.571178V1-supplement-3.pdf]

bioRxiv preprint doi: https://doi.org/10.1101/2023.12.13.571178; this version posted December 14, 2023. The copyright holder for this preprint (which was not certified by peer review) is the author/funder, who has granted bioRxiv a license to display the preprint in perpetuity. It is made available under aCC-BY 4.0 International license.

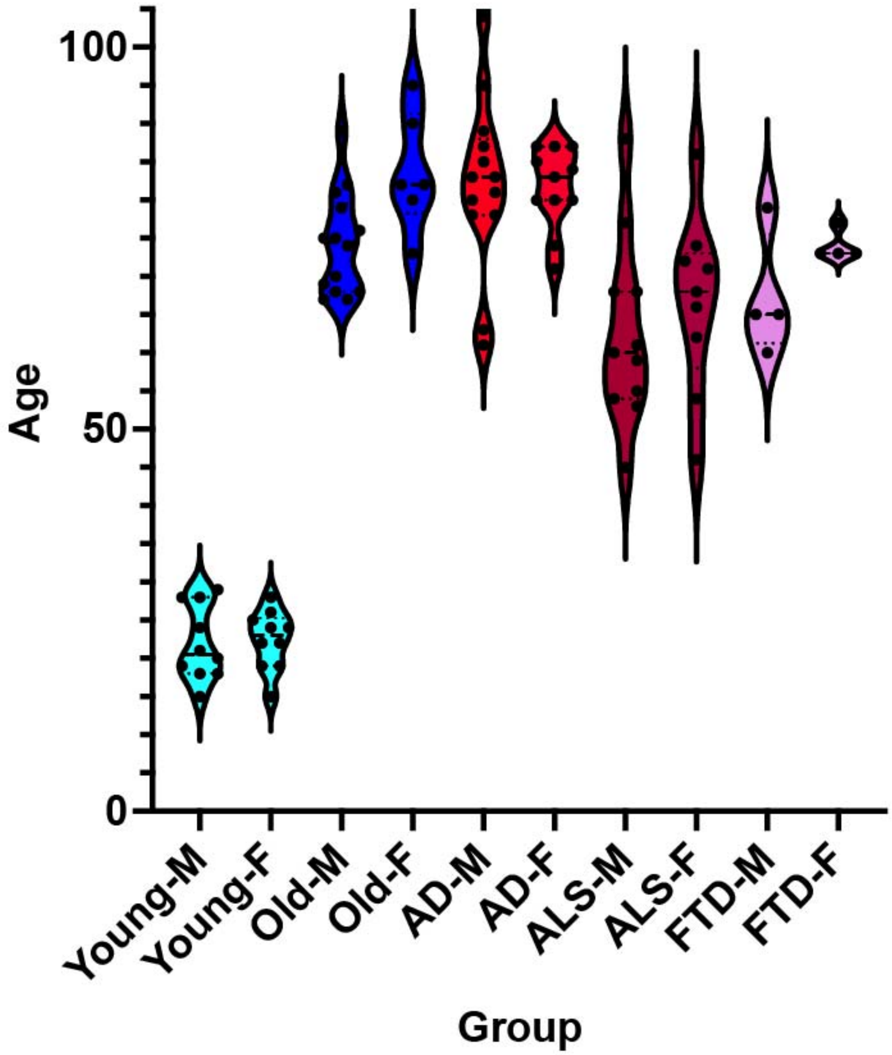

SI Figure 1. Age and sex distribution of cortical samples used.
Plots show the age of individual sample and their distribution.

bioRxiv preprint doi: https://doi.org/10.1101/2023.12.13.571178; this version posted December 14, 2023. The copyright holder for this preprint (which was not certified by peer review) is the author/funder, who has granted bioRxiv a license to display the preprint in perpetuity. It is made available under aCC-BY 4.0 International license.

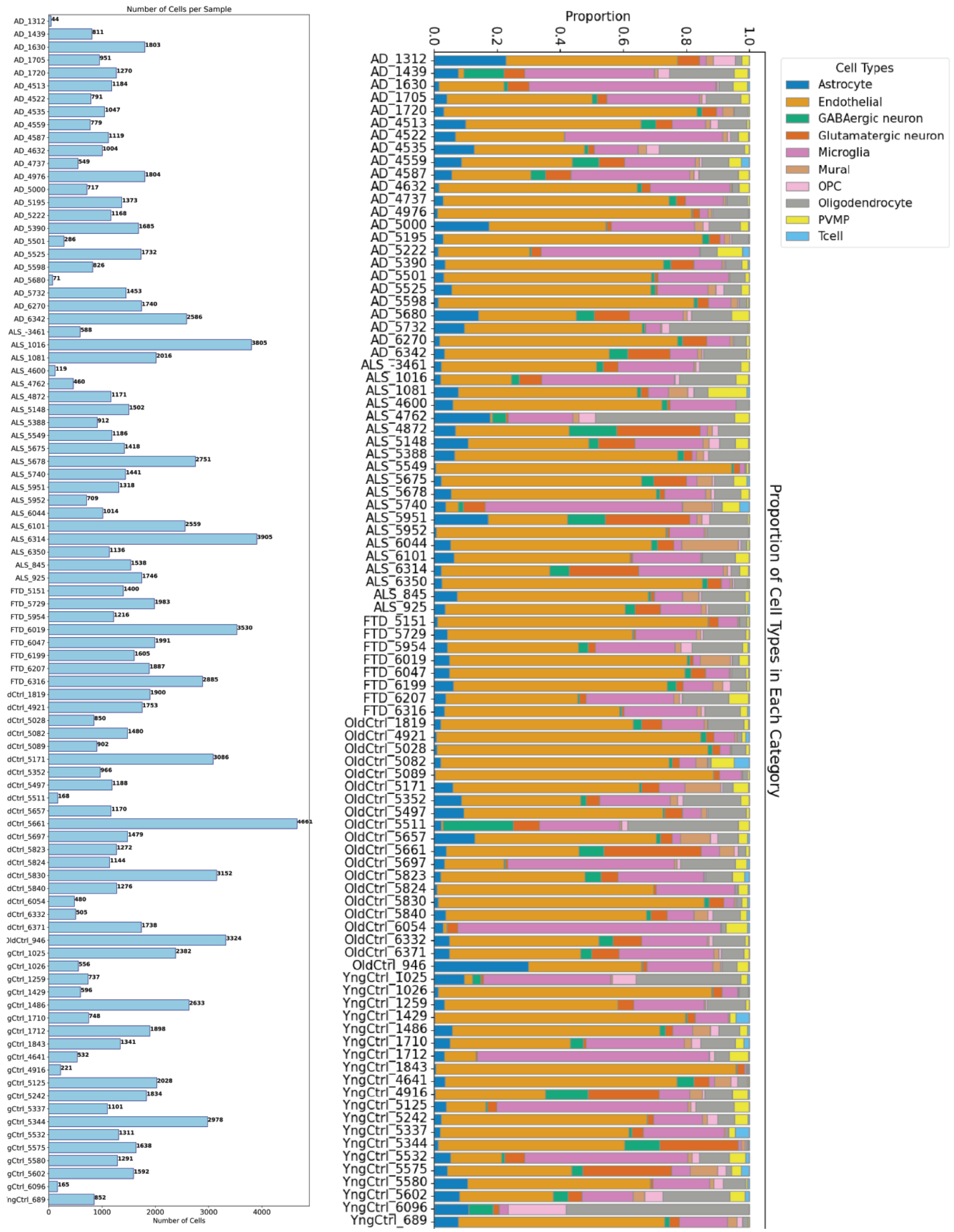

SI Figure 2. Number of nuclei and distribution of cell types in data.

Sample ids are shown on the y-axis, and plots show total nuclei (with count), endothelial nuclei (with count), and distribution of cell types (key in top left).

bioRxiv preprint doi: https://doi.org/10.1101/2023.12.13.571178; this version posted December 14, 2023. The copyright holder for this preprint (which was not certified by peer review) is the author/funder, who has granted bioRxiv a license to display the preprint in perpetuity. It is made available under aCC-BY 4.0 International license.

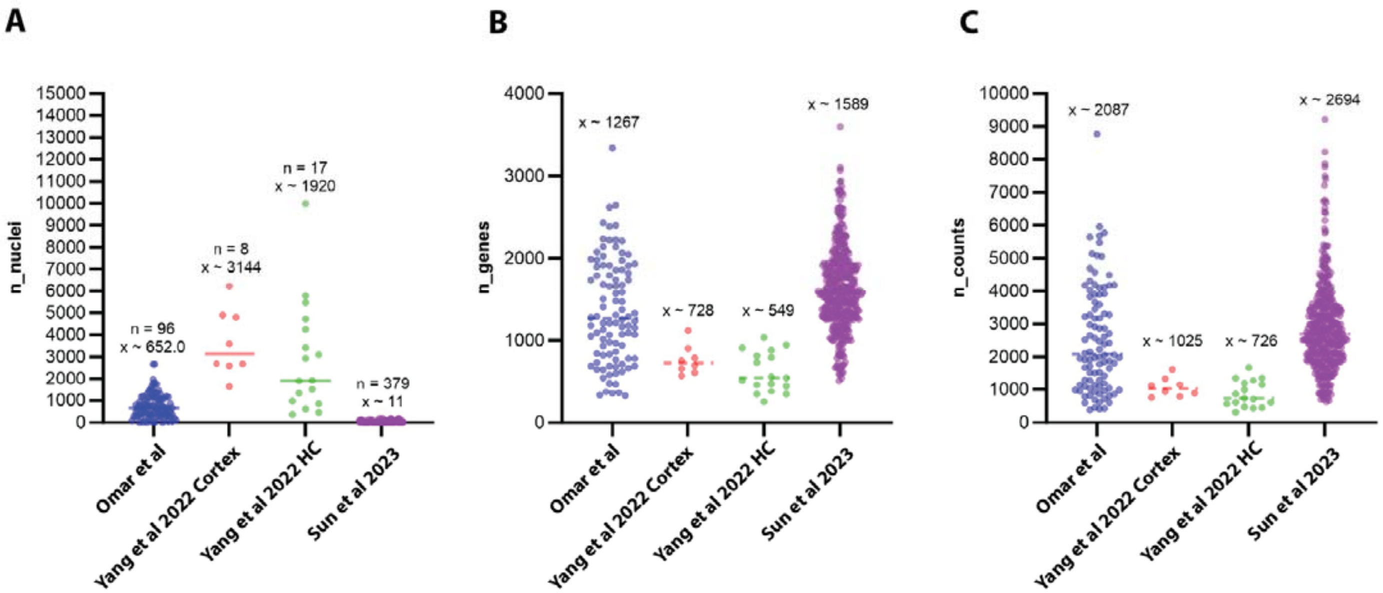

SI Figure 3. Comparison of gene counts and UMI with prior data sets.
Comparison of number of nuclei per donor, and number of genes and counts per donor in data described here, relative to recently published data sets on brain vasculature and endothelium.

bioRxiv preprint doi: https://doi.org/10.1101/2023.12.13.571178; this version posted December 14, 2023. The copyright holder for this preprint (which was not certified by peer review) is the author/funder, who has granted bioRxiv a license to display the preprint in perpetuity. It is made available under aCC-BY 4.0 International license.

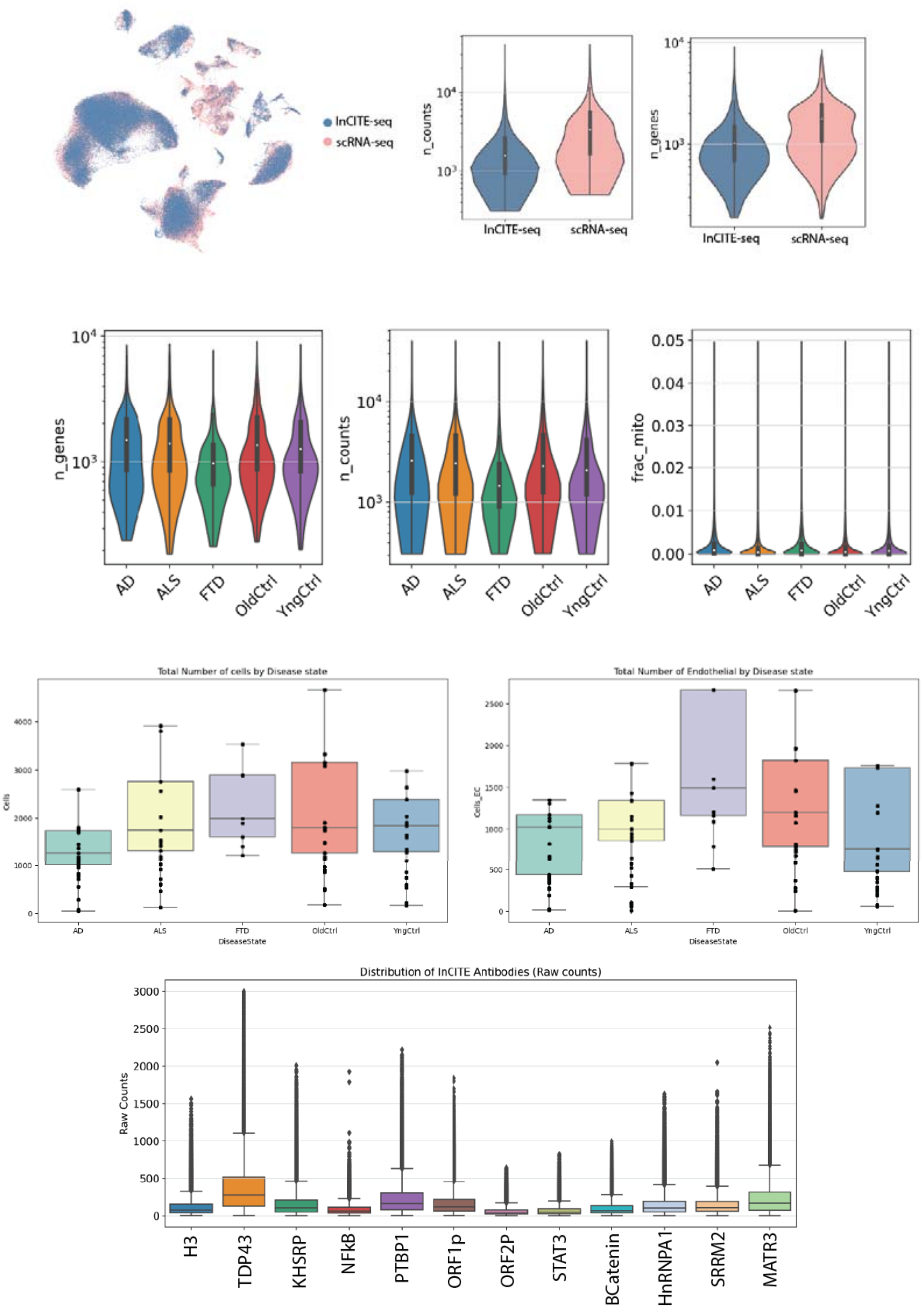

SI Figure 4. Comparative gene counts and umi in single nuclei data (with and without inCITE-Seq)
UMAP showing overlap between single nuclei data obtained from standard scRNA-Seq and inCITE-Seq preparations. UMI counts per cells and gene counts per cell are shown overlap, and by subcategory, along with mitochondrial RNA counts. Plots show the total number of cells by disease state in each method.

bioRxiv preprint doi: https://doi.org/10.1101/2023.12.13.571178; this version posted December 14, 2023. The copyright holder for this preprint (which was not certified by peer review) is the author/funder, who has granted bioRxiv a license to display the preprint in perpetuity. It is made available under aCC-BY 4.0 International license.

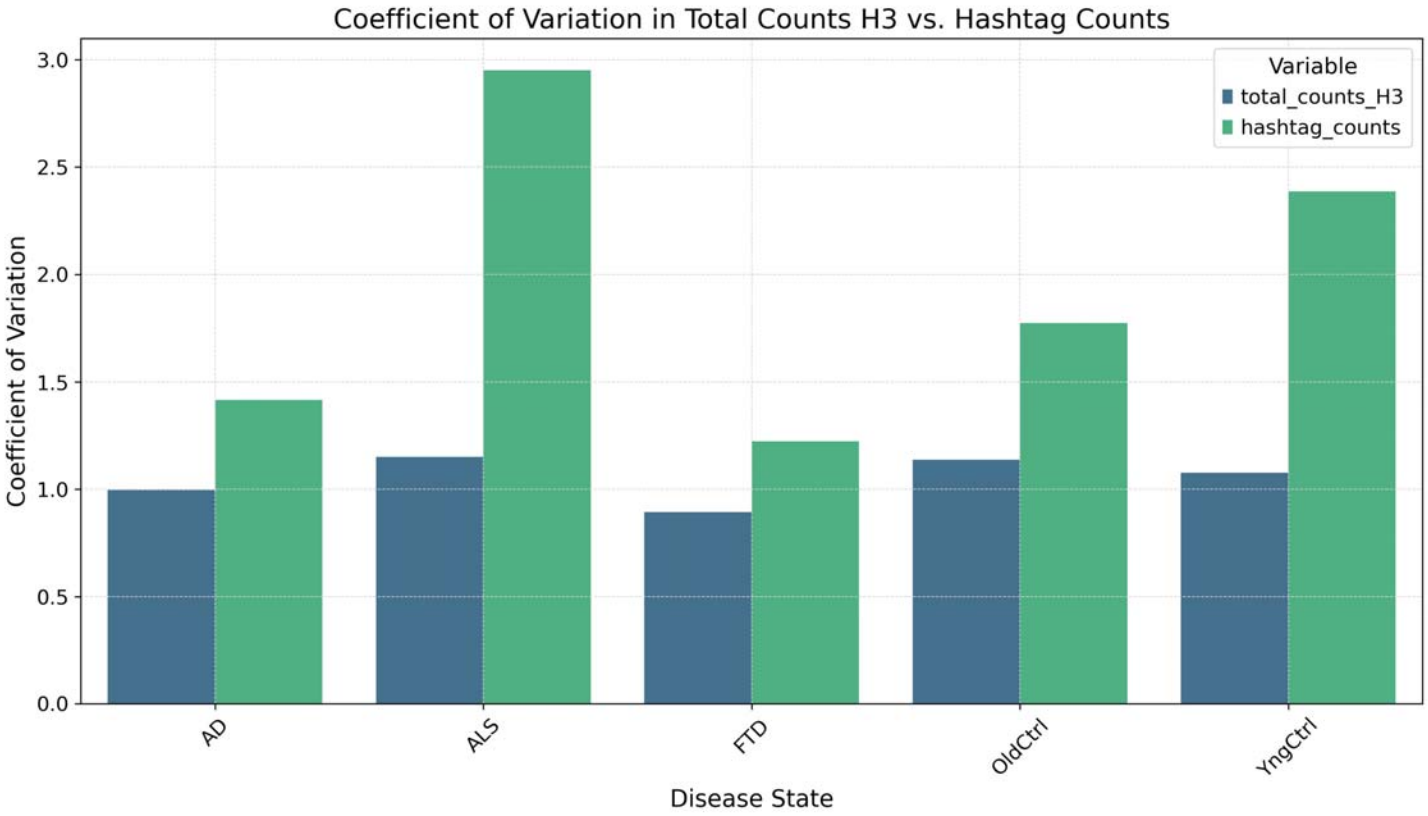

SI Figure 5. Coefficient of variation in the levels of histone and nuclear pore hashtags between nuclei by disease state. Violin plot shows coefficient of variation in levels of histone and nuclear pore hashtags across nuclei within each disease group.

bioRxiv preprint doi: https://doi.org/10.1101/2023.12.13.571178; this version posted December 14, 2023. The copyright holder for this preprint (which was not certified by peer review) is the author/funder, who has granted bioRxiv a license to display the preprint in perpetuity. It is made available under aCC-BY 4.0 International license.

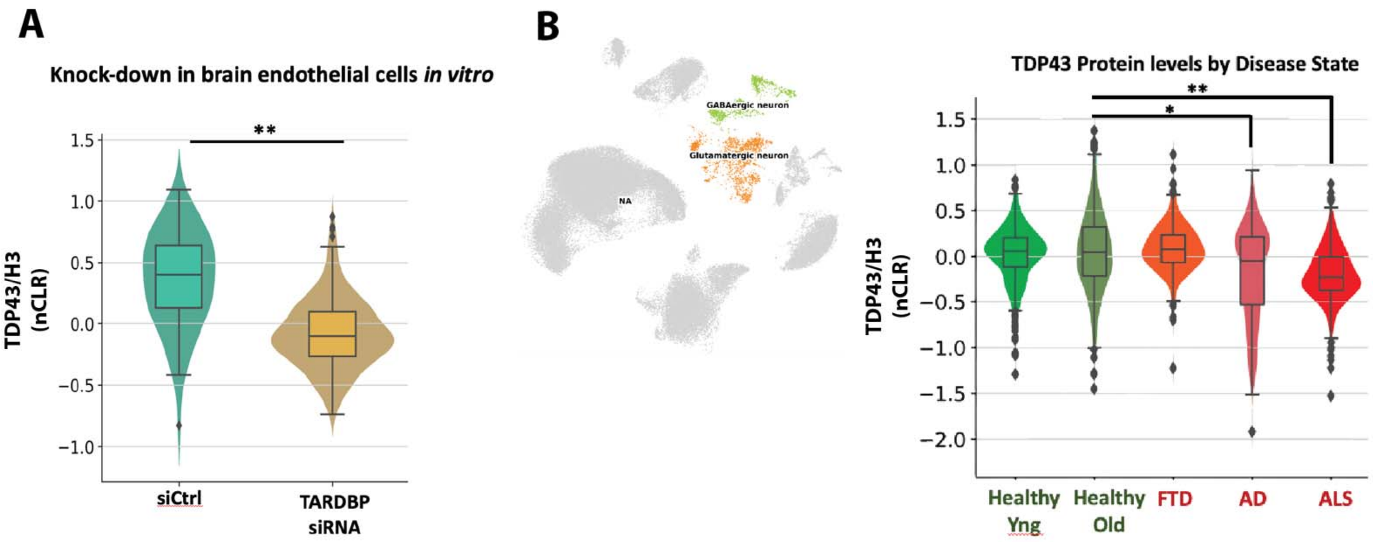

SI Figure 6. Validation of TDP-43 inCITE analysis

(A) siTDP-43 knockdown in HBEC5i human brain endothelial cell line, compared to siControl treated HBEC5i cells, and analyzed in an inCITEseq experiment along with a standard brain nuclei preparation. (B) UMAP showing neuronal clusters analyzed, and violin plots showing the levels of nuclear TDP-43 protein (relative to nuclear H3) by disease state in these neuronal clusters.

bioRxiv preprint doi: https://doi.org/10.1101/2023.12.13.571178; this version posted December 14, 2023. The copyright holder for this preprint (which was not certified by peer review) is the author/funder, who has granted bioRxiv a license to display the preprint in perpetuity. It is made available under aCC-BY 4.0 International license.

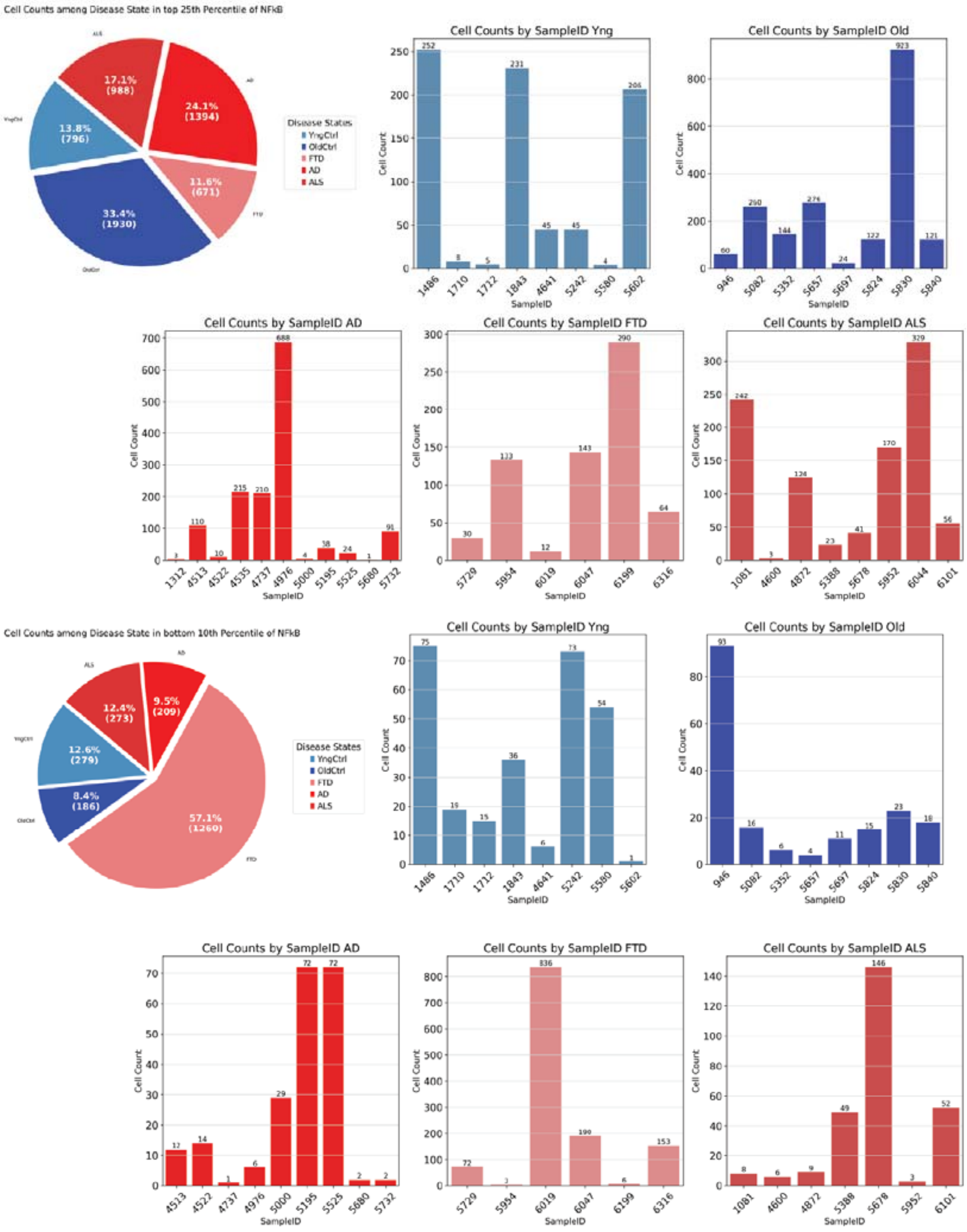

SI Figure 7. Composition of samples in extremes of p65/NFkB

Pies chart bar plots showing the proportion of cells in the top 25th percentile of p65/NFkB:H3 and the bottom 10th percentile of p65/NFkB:H3.

bioRxiv preprint doi: https://doi.org/10.1101/2023.12.13.571178; this version posted December 14, 2023. The copyright holder for this preprint (which was not certified by peer review) is the author/funder, who has granted bioRxiv a license to display the preprint in perpetuity. It is made available under aCC-BY 4.0 International license.

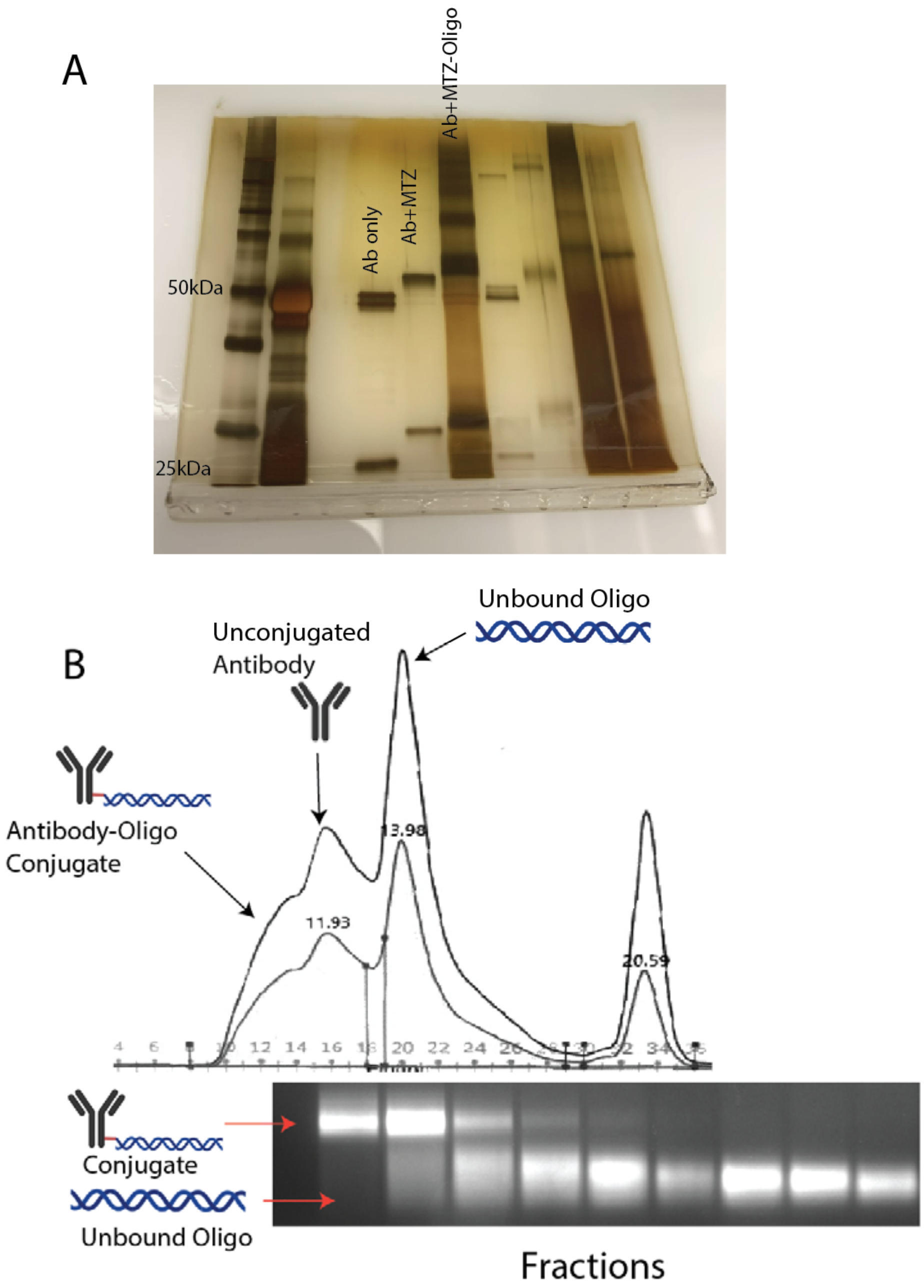

SI Figure 8. Size-exclusion purification of oligo conjugated antibodies.
(A) Reduced and silver stained gel showing the step wise addition of click chemistry adduct (MTZ) and oligo nucleotide (MTZ-Oligo). (B) Oligo-conjugated antibody cleanup via size exclusion chromatography. Top, a representative size exclusion chromatogram recorded with dual absorption wavelengths (260 and 280 nm). Conjugated antibodies, having the largest molecular weights, were eluted first from the column, followed by the free antibodies. Unconjugated oligonucleotides were the last to be eluted from the column. Bottom, fractions from the column were analyzed by an agarose gel stained with SybrGold showing the presence of free and conjugated oligonucleotides.
